# Pulmonary lymphangitis carcinomatosis: A peculiar presentation clustering in MET‐amplified gastric cancer

**DOI:** 10.1002/cam4.6575

**Published:** 2023-09-29

**Authors:** Zhening Zhang, Yiyi Yu, Tong Xie, Changsong Qi, Xiaotian Zhang, Lin Shen, Zhi Peng

**Affiliations:** ^1^ State Key Laboratory of Holistic Integrative Management of Gastrointestinal Cancers, Beijing Key Laboratory of Carcinogenesis and Translational Research, Department of Gastrointestinal Oncology Peking University Cancer Hospital & Institute Beijing China; ^2^ Fudan Zhongshan Cancer Center Zhongshan Hospital Fudan University Shanghai China

**Keywords:** gastric cancer, heterogeneity, MET amplification, pulmonary lymphangitis carcinomatosis, targeted therapy

## Abstract

**Background:**

The clinicopathological features of MET‐amplified gastric cancer (GC) and real‐world data on the efficacy of MET‐targeted therapies remain unknown. Pulmonary lymphangitis carcinomatosis (PLC) is a peculiar manifestation of GC, whose management has not been thoroughly described.

**Methods:**

This study analyzed patients diagnosed with MET‐amplified GC or GC with PLC at any time point of the disease course from 2011 to 2021 in two centers. Clinicopathological features and survival outcomes of MET‐amplified GC were analyzed. The clinical and molecular implications of GC with PLC were discussed.

**Results:**

Fifty‐eight patients with MET‐amplified GC and 20 patients with GC accompanied by PLC were finally enrolled for analysis (including 13 overlapped patients). GC with PLC was more common in female patients (*p* = 0.010), diagnosed at a younger age (*p* = 0.002), presented with a higher baseline ECOG PS (*p* = 0.016), and was more likely to develop lung metastasis (*p* < 0.001), and serous effusion (*p* = 0.026) than GC without PLC. Patients with primary MET‐amplified GC had a worse prognosis than those with secondary MET‐amplified GC (*p* = 0.005). The application of anti‐MET therapy was associated with numerically prolonged survival, but the association was not statistically significant (*p* = 0.07). MET amplification was concentrated in patients with PLC, in which anti‐MET therapies elicited a high response rate.

**Conclusions:**

MET‐targeted therapies are efficacious in real‐world populations with MET‐amplified GC. Patients with PLC have distinct clinical and molecular features and might benefit from MET‐targeted therapies.

## 1 INTRODUCTION

In 2018, gastric cancer (GC) was the fifth most common malignancy (1,034,000 new cases, 5.7% of all cancers) and the third leading cause of cancer‐related death worldwide (783,000 deaths, 8.2% of all cancer‐related deaths).^1^ The prognosis of patients with late‐stage GC remains poor. In this context, the development of novel therapies for metastatic or recurrent GC is warranted.

There is increasing enthusiasm for exploring avenues for molecular targeted therapies of GC after the encouraging results of trastuzumab in human epidermal growth factor receptor 2 (HER2)‐positive GC patients presented by the ToGA trial.^2^ Additional molecular targets are now being investigated, including vascular endothelial growth factor receptor 2 (VEGFR2), fibroblastic growth factor receptor (FGFR), and mesenchymal to epithelial transition (MET).^3^, ^4^, ^5^, ^6^, ^7^, ^8^

C‐Met, a receptor tyrosine kinase (RTK) encoded by the MET gene, and its ligand hepatocyte growth factor (HGF) are targets receiving extensive attention.^9^ Aberrantly activated HGF/MET signaling is involved in cancer progression and could predict poor prognosis in metastatic GC.^8^, ^10^, ^11^, ^12^ MET overexpression (constituting 50%–60% of advanced GC), MET gene mutations (typically exon 14 skipping mutation) and amplification (constituting 4%–6% of advanced GC) are the most common forms of HGF/MET pathway alterations, yet their clinical implications are varied.^13^, ^14^, ^15^ In addition, MET amplification contains two forms: (a) primary amplification is defined as activated MET signaling in treatment‐naïve tumors; (b) secondary amplification refers to newly detected MET overactivation in treated tumors (primary and/or metastatic sites).^16^ It has been suggested in previous research that anti‐MET monoclonal antibodies fail to improve survival in patients with MET‐overexpressing GC.^17^, ^18^ A phase II, multicenter, randomized controlled trial in France found that adding rilotumumab to standard chemotherapy did not bring any benefits to GC patients.^19^ Despite this, MET tyrosine kinase inhibitors (TKIs), such as crizotinib, may result in a positive response in a specific group of screened patients with MET‐amplified GC.^20^, ^21^, ^22^, ^23^ These findings suggest that de novo MET amplification could be a pivotal driver of oncogenesis and a druggable target. However, the baseline characteristics of patients could be tremendously different in clinical trials versus those in the real‐world. This triggers an interest in investigating the authentic efficacy of anti‐MET therapies in unselected populations, which has never been reported.

Pulmonary lymphangitis carcinomatosis (PLC) is a peculiar pattern of lung metastasis featuring lymphatic permeation and lymph capillary infiltration by tumor cells, which portends a miserable prognosis. Previous studies have indicated that breast, gastric, and lung cancers are the most common malignancies that give rise to PLC, while the pathogenic mechanisms of PLC are not understood.^24^ In addition, patients with PLC generally succumb to poor physical condition and thus are often excluded from clinical trials. Judicious management of PLC remains an unmet need.

We sought to investigate the clinicopathological and prognostic features of MET‐amplified GC. Our study also revealed that MET amplification is a molecular event enriched in GC with PLC, which represents a distinct subtype showing responses to molecular targeted therapies.

## 2 METHODS

### 2.1 Study population

In our two‐center retrospective study, eligible patients were diagnosed with recurrent or metastatic GC that was either MET‐amplified or manifested with PLC at any time point of the disease course from 2011 to 2021 in two medical centers. Tumor samples were generated from all patients by endoscopy, surgery, or needle biopsy at primary and/or secondary tumor sites. Tissues were fixed with formalin and paraffin‐embedded in blocks, which were prepared at the time of biopsy. Patients with available baseline characteristics and clinical data were included in the analysis. All patients were followed until October 2021.

### 2.2 Confirmation of MET status and PLC

MET gene copy numbers in all tumor samples were detected by next‐generation sequencing (NGS). NGS analysis was conducted by Genecast, Inc., a College of American Pathologists (CAP)‐accredited laboratory. A customized 769‐gene panel (Genecast) was employed to detect point mutations, indels, fusions, rearrangements, and CNVs. Targeted sequencing was performed on the NovaSeq 6000 platform (Illumina). The cutoff for MET amplification was 2.0 times (averaged MET copy number in tumor tissue normalized by normal comparator). The PLC was determined by two independent radiologists based on chest computed tomography (CT) scans. Typical radiological findings include smooth or nodular thickening of interlobular septa, peribronchovascular interstitium, polygonal arcades with thickened limbs, and ground glass opacities. Clinical manifestations (e.g., progressive dyspnea, cough, and tachypnea) also assisted in the diagnosis.

### 2.3 Immunohistochemistry (IHC)

HER2, programmed death ligand 1 (PD‐L1), and mismatch repair (MMR) status were evaluated through IHC. HER2 expression was analyzed using the anti‐HER‐2 (4B5) rabbit monoclonal primary antibody (Ventana, U.S.). HER2 positivity was defined as IHC 3+ or both IHC 2+ and FISH positive. IHC of MLH1, MSH2, MSH6, and PMS2 was performed with a BenchMark ULTRA slide processing system (Ventana, U.S.) according to the manufacturer's instructions. Tumors were defined as deficient mismatch repair (dMMR) only if at least one of the proteins was absent of staining in the tumor cells; otherwise, proficient mismatch repair (pMMR) was deemed. PD‐L1 staining was performed using a 22C3 pharmDx kit (Dako, Denmark). A combined positive score (CPS, defined as the total number of tumor cells and immune cells stained positive divided by the number of viable tumor cells, then multiplied by 100) of 1 or higher was considered PD‐L1 positive.

### 2.4 In situ hybridization (ISH)

HER2 amplification was detected by fluorescence in situ hybridization (FISH) using a PathVysion HER‐2 DNA probe kit (Abbott Laboratories, U.S.). Cases with a HER2:centromere 17 ratio >2.0 were considered FISH positive. The presence of EBV infection was determined by ISH using probes against Epstein–Barr virus‐encoded RNAs (EBER); (Leica Biosystems). Specimens with dark blue staining in tumor cell nuclei were deemed EBV‐positive.

### 2.5 Statistical analysis

Statistical analyses were performed using SPSS software (v.24.0; IBM Corporation, U.S.) and Prism (v.9; GraphPad Software, U.S.). Categorical variables were evaluated using the chi‐square test or Fisher's exact test, as appropriate. Survival curves were plotted using the Kaplan–Meier method, and survival rates were compared between groups using the log‐rank test. Cox multivariate regression analyses were used to determine factors that affect survival outcomes. Overall survival (OS) was calculated from the time of diagnosis until death or the last observation for surviving patients. Progression‐free survival (PFS) was defined as the interval from the start of therapy to treatment discontinuation for disease progression or death of any cause. All tests were two‐sided, and differences were considered significant when *p* < 0.05.

## 3 RESULTS

### 3.1 Characteristics of patients with MET‐amplified GC according to PLC manifestation

A total of 937 patients diagnosed with stage IV GC were included for eligibility screening. Of these, 65 patients were eligible and had available clinicopathological information. Fifty‐eight patients with MET gene‐amplified GC and 20 patients with GC accompanied by PLC were finally enrolled for analysis (including 13 overlapped patients). A flow diagram is shown in Figure S1. Patient demographics and tumor characteristics are summarized in Table S1. Among patients with MET‐amplified GC (*n* = 58, accounted for 6.2% of the total patients), the median age at diagnosis was 53 years (range, 23–84 years), with a male preponderance. Twenty‐eight out of 58 (48.3%) patients had serous effusion. Liver, bone, and lung metastasis occurred in 21 (36.2%), 15 (25.9%), and 21 (36.2%) patients, respectively. GC with PLC was more common in females (42.9% vs. 10.8%, *p* = 0.010), diagnosed at a younger age (median age, 44 vs. 56 years, *p* = 0.002), and presented with a higher baseline ECOG PS (≥2 points, 46.2% vs. 17.8%, *p* = 0.016) than GC without PLC. Lung metastasis and serous effusion were more frequent in GC with PLC than in GC without PLC (84.6% vs. 22.2%, *p* < 0.001; 61.5% vs. 44.4%, *p* = 0.026). GC with PLC accounted for a higher percentage of bone metastasis and poorly differentiated histology (38.5% vs. 22.2%, *p* = 0.091; 92.3% vs. 71.1%, *p* = 0.084) than GC without PLC, though the differences were not significant. For molecular features, the average MET gene copy number was 7.4 (range, 2.1–29.0). No difference was found in MET gene copy number according to PLC (7.2 vs. 8.0, *p* = 0.76). One out of 13 GC with PLC and 6 out of 45 GC without PLC were HER2 positive. All patients with detectable pathological specimens had pMMR and EBV‐negative tumors. Thirty‐nine percent of patients (15/38) had PD‐L1‐positive GC, with CPS ranging from 1 to 98.

### 3.2 Comparisons of therapeutic outcomes determined by PLC manifestation

Overall, 58 MET‐amplified GC patients were included for analysis, of which 41.4% (24/58) received ≥3 lines of therapies. Twenty‐eight patients received anti‐MET TKIs, of which 13 (46.3%) achieved PR, 4 (14.3%) achieved SD, and 3 (10.7%) achieved non‐CR non‐PD for the best response. The median duration of response was 3.0 months (range, 1.5–14.0 months). Most of the patients received anti‐MET therapies as first‐ or second‐line treatment (20/28). In our cohort, patients with PLC received more lines of therapies, with 68.8% (11/16) undergoing ≥3 lines of therapies. In addition, patients with MET‐amplified GC and PLC underwent more intensive treatments, including a higher frequency of anti‐MET therapies, antiangiogenic therapies, and immunotherapies (*p* = 0.038; *p* = 0.029; *p* = 0.016, respectively). (Table 1).

**TABLE 1:** Treatment and survival of patients with MET‐amplified GC according to PLC.

	Total cases (*n* = 58)	MET‐amplified GC with PLC (*n* = 13)	MET‐amplified GC without PLC (*n* = 45)	*p*‐Value
*Variables*				
Taxane‐based chemotherapy				
Yes	38	8	30	1.000
No	20	5	15	
Immunotherapy				
Yes	24	10	14	0.016
No	34	3	31	
Antiangiogenic therapy				
Yes	19	7	12	0.029
No	39	6	33	
Anti‐MET therapy				
Yes	28	10	18	0.038
No	30	3	27	

### 3.3 Analysis of survival outcomes of MET‐amplified GC patients

**FIGURE 1:**
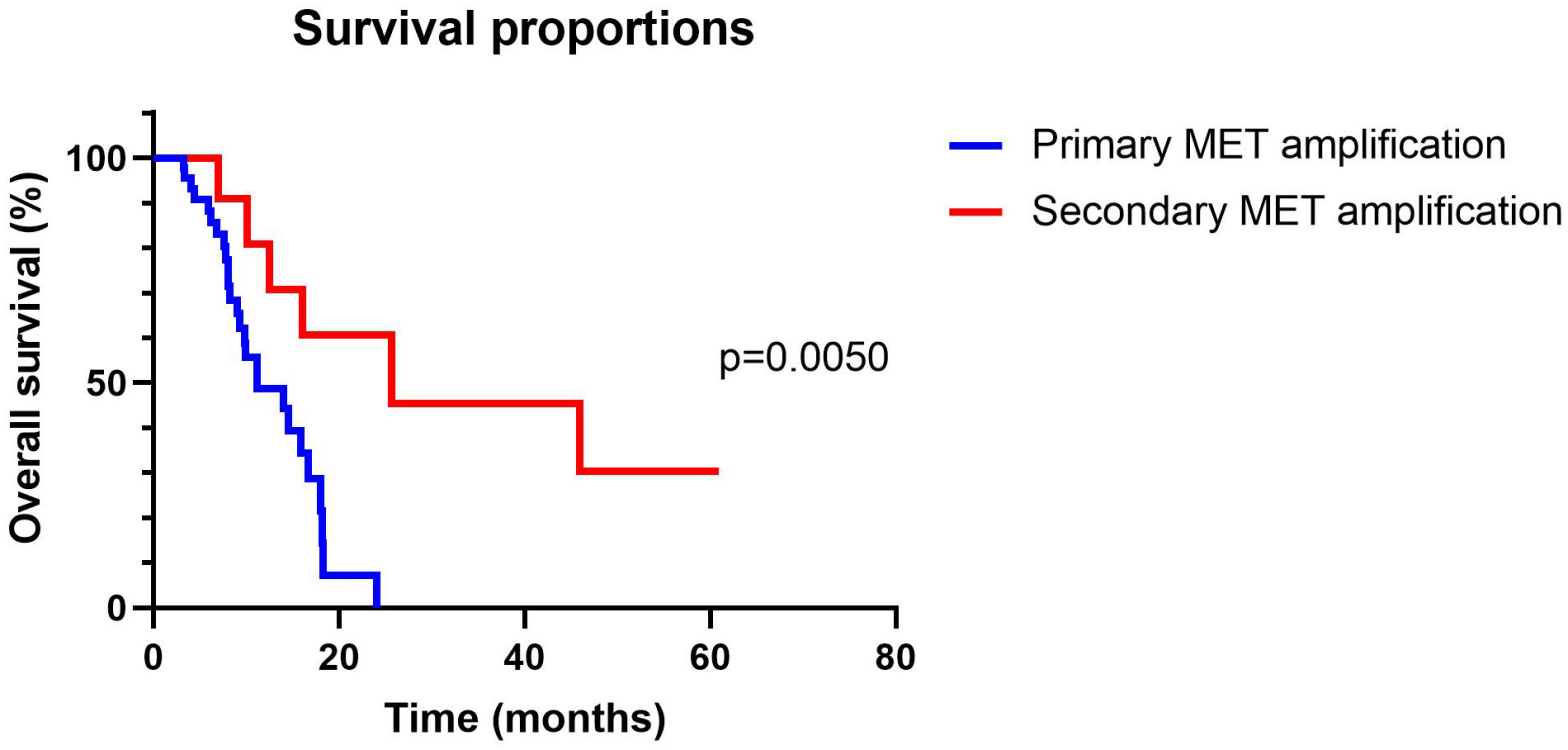
Survival curves of MET‐amplified GC patients according to different amplification types.

**FIGURE 2:**
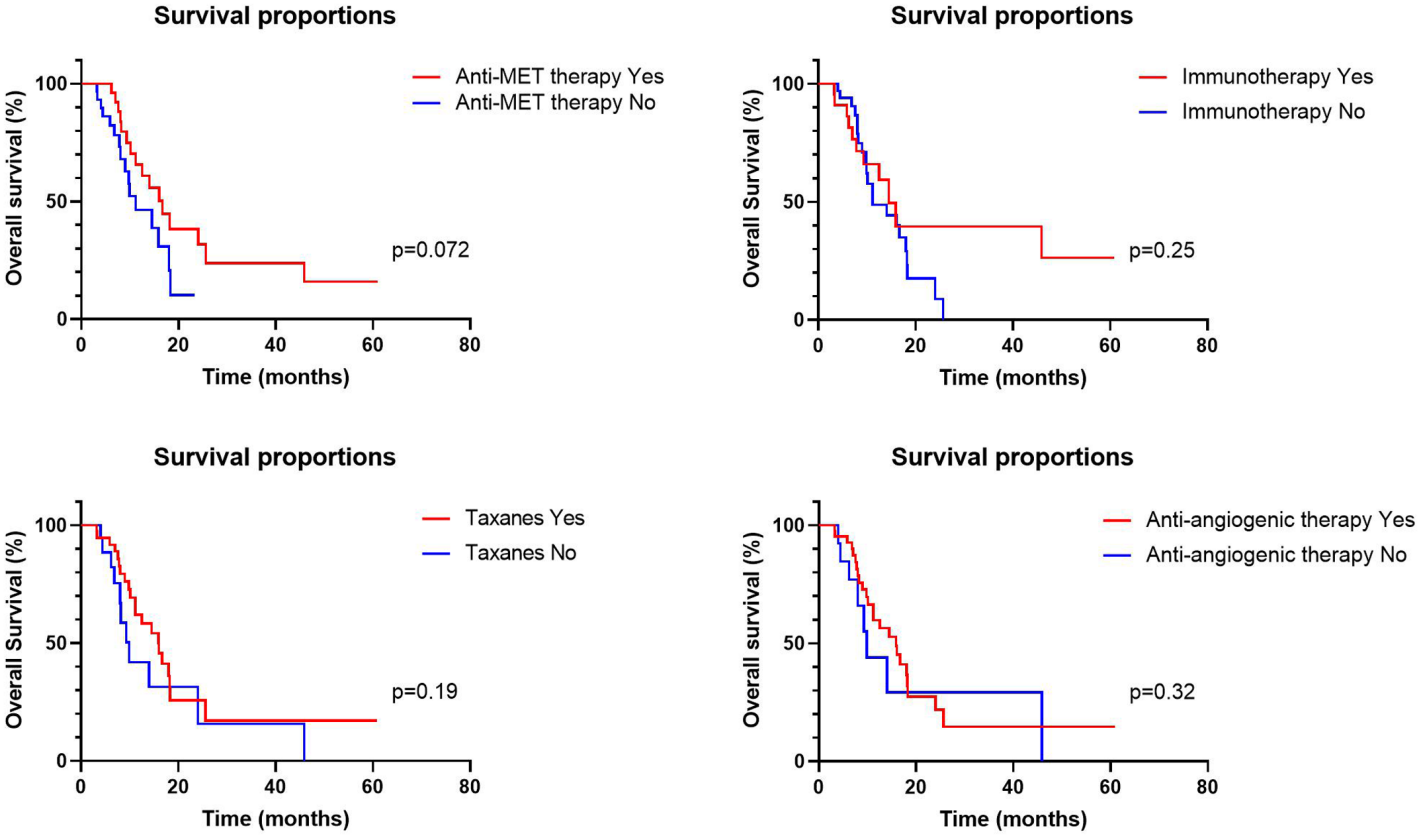
Survival curves of MET‐amplified GC patients according to different therapies.

Survival curves were plotted and compared according to tumor metastatic sites, treatment regimens, clinicopathological parameters, and MET amplification forms. In our cohort, 45 patients had tumors with primary MET amplification, while 13 patients had tumors with secondary MET amplification. Patients with primary MET‐amplified GC presented shorter OS than those with secondary MET‐amplified GC (11.2 vs. 25.6 months, *p* = 0.005); (Figure 1). The presence or absence of liver, bone, lung, or peritoneum metastasis were not correlated with OS (Figure S2). Survival outcome was not different between patients with or without PLC regarding HER2 status or PD‐L1 expression. The onset of thorax or peritoneum effusion was not correlated with worse survival (Figure S3). Upon further analysis of the correlation between the therapeutic regimen and OS, we found that patients who received anti‐MET therapy had prolonged survival numerically (16.7 vs. 11.2 months), while a significant difference was not seen (*p* = 0.07); (Figure 2). The usage of taxane‐based chemotherapy, immunotherapy, or antiangiogenic therapies was not associated with OS.

In Cox regression analysis, age, gender, and prognostic factors with *p* < 0.15 in univariate analysis (including ECOG PS, presence of PLC, presence of lung metastasis, primary MET amplification, and anti‐MET therapy) were added into the multivariate model. Only secondary MET amplification was a predictive factor of a prolonged OS with statistical significance (*p* = 0.007; hazard ratio, 0.017; confidence intervals, 0.001–0.322).

### 3.4 Characteristics and treatments of patients with PLC

A total of 20 patients were diagnosed with PLC and included for analysis. The median age at diagnosis was 42.5 years (range 23–77 years). Eleven out of 20 patients were female. Ten patients had an ECOG PS of 0–1, and the other 10 patients had an ECOG PS of 2–3. Seventeen patients had tumors with assessable MET gene status, of which 13 (76.5%) were confirmed MET‐amplified and otherwise nonamplified. The genomic variation information of 17 patients with PLC is shown in Figure S4. Four and seven out of 20 patients were HER2 positive and PD‐L1 positive, respectively. All included patients had EBV‐negative and pMMR tumors. Tumor mutation burden (TMB) ranged from 1.93 to 14.01 mutations per megabase, denoting that all patients with PLC in our cohort had TMB‐low tumors. Thirteen out of 20 patients (65.0%) manifested serous effusion, suggesting its high incidence rates in GC with PLC. The lung, nonregional lymph nodes, liver, adrenal glands, ovary, and bone were common metastatic sites. Other clinicopathological characteristics are presented in Table 2.

**TABLE 2:** Clinicopathological and molecular properties of patients with PLC.

No.	Sex/Age	ECOG PS	MET status	HER2 status	PD‐L1 status	EBER status	MMR status	Histology/differential degree/Lauren classification	Location of the primary tumor	Metastatic sites	Serous effusion
1	F/23	3	Amplification 5.7 times	Negative	CPS = 5	Negative	pMMR	AC + SRC/poor/diffuse	Body	LNM, lung, peritoneum, adrenal gland, ovary, bone	Hydrothorax, hydroperitoneum, hydropericardium
2	F/38	0	Amplification 9.9 times	Positive (IHC 3+)	CPS < 1	Negative	pMMR	SRC	Antrum	LNM, lung, bone	None
3	M/42	2	Amplification 20.1 times	Negative	CPS < 1	Negative	pMMR	AC + SRC/poor/diffuse	Antrum	LNM, lung, peritoneum, bone	Hydrothorax
4	F/41	1	Amplification 7.4 times	Negative	CPS < 1	Negative	pMMR	AC + SRC/poor/diffuse	Body	LNM, lung, bone, ovary	Hydrothorax
5	F/26	1	No amplification	Negative	CPS < 1	Negative	pMMR	SRC	Body	LNM, lung, bone, spleen, pancreas, ovary	Hydrothorax, hydroperitoneum
6	M/37	2	No amplification	Negative	CPS = 10	Negative	pMMR	SRC	Body	LNM, lung, brain	Hydrothorax, hydroperitoneum
7	M/54	3	NA	Positive (IHC 3+)	CPS = 5	Negative	pMMR	AC/moderate/mixed	Body	LNM, lung	Hydrothorax, hydropericardium
8	F/58	2	No amplification	Negative	CPS < 1	Negative	pMMR	AC + SRC/poor/diffuse	Body, antrum	LNM, lung, ovary, bone, BM	Hydrothorax
9	M/59	1	No amplification	Negative	CPS < 1	Negative	pMMR	AC/moderate/diffuse	Cardia	LNM, bone, liver	None
10	F/43	1	NA	Negative	CPS < 1	Negative	pMMR	AC + SRC/poor/diffuse	Body	LNM, lung, peritoneum, ovary	None
11	M/62	2	NA	Negative	CPS = 50	Negative	pMMR	AC + SRC/poor/mixed	Angle	LNM, lung, liver, bone, adrenal gland	Hydrothorax
12	M/62	1	Amplification	Negative	CPS < 1	Negative	pMMR	AC + SRC/poor/diffuse	Antrum	LNM, lung, bone, pancreas, liver, adrenal gland	None
13	M/77	1	Amplification 6.5 times	Negative	CPS = 10	Negative	pMMR	AC + NEC/poor/intestinal	Cardia	LNM, lung, bone	None
14	M/33	1	Amplification 7.3 times	Negative	CPS < 1	Negative	pMMR	AC/poor/diffuse	Body	LNM, lung, liver	None
15	F/38	2	Amplification 6.3 times	Negative	CPS < 1	Negative	pMMR	AC/moderate/intestinal	Antrum	LNM, lung, bone, liver, adrenal gland, ovary	None
16	F/50	2	Amplification 13.8 times	Negative	CPS < 1	Negative	pMMR	AC/poor/diffuse	Body	LNM, lung, bone, pericardium, ovary, peritoneum, brain	Hydrothorax, hydroperitoneum
17	F/42	2	Amplification 6.7 times	Negative	CPS < 1	Negative	pMMR	AC/poor/diffuse	Body	LNM, lung, bone, pericardium, ovary, peritoneum	Hydrothorax, hydroperitoneum
18	F/32	2	Amplification 9.8 time	Negative	CPS < 1	Negative	pMMR	AC/poor/diffuse	Antrum	LNM, lung, peritoneum, ovary	Hydroperitoneum
19	M/55	1	Amplification 2.5 times	Negative	CPS = 98	Negative	pMMR	AC/poor/diffuse	Body	LNM, lung, liver, spleen, pancreas, peritoneum	Hydroperitoneum
20	F/45	1	Amplification 2.1 times	Negative	CPS = 2	Negative	pMMR	AC/poor/diffuse	Body	LNM, lung	Hydroperitoneum

Abbreviations: AC, adenocarcinoma; BM, bone marrow; CPS, combined positive score; EBER, Epstein–Barr virus‐encoded RNAs; ECOG, Eastern Cooperative Oncology Group; FISH, fluorescence in situ hybridization; HER2, human epidermal receptor 2; IHC, immunohistochemistry; LNM, lymph node metastasis; MMR, mismatch repair; NA, not available; NEC, neuroendocrine carcinoma; PD‐L1, programmed death ligand 1; PS, performance status; SRC, signet ring cell carcinoma.

Eleven patients received ≥3 lines of treatment for PLC. Ten patients received anti‐MET therapies. Regarding drug types, 10 patients received anti‐MET therapy with crizotinib, one of whom also received volitinib. Owing to the absence of measurable lesions in a few cases, therapeutic responses were determined from two perspectives (both symptomatically and radiologically). Fourteen patients rapidly alleviated their symptoms (e.g., cough, dyspnea, and fatigue) or obtained radiographical remission after systemic therapies. Figure S5 Displays a representative CT image of PLC from a MET‐amplified GC patient. In particular, seven out of 10 patients with MET‐amplified GC achieved a PR after anti‐MET therapies, among which the median PFS was 6.0 months. Nearly half of the patients (9/20) had stabilized PLCs but developed new lesions (Table 3). The median OS of patients with PLC was 11.2 months, and the median interval between the first onset of PLC and PLC progression was 2.5 months.

**TABLE 3:** Treatment and responses of patients with pulmonary lymphangitis carcinomatosis.

No.	Treatment of PLC	Best clinical response	TTP (m)	Progression site	Anti‐MET treatment	BOR	PFS (m)
1	3rd line: docetaxel + capecitabine + cisplatin (ip)	SD, PLC relieved (symptomatic)	NP	NA	No	NA	NA
2	5th line: trastuzumab + capecitabine + oxaliplatin 6th line: crizotinib + trastuzumab	PD/PD, PLC relieved (radiographic)	NA	LNM, bone, peritoneum	Yes	PD	2.0
3	2nd line: albumin paclitaxel + S‐1 3rd line: crizotinib	PD/PR, PLC relieved (symptomatic)	1.5	Lung	Yes	PR	1.5
4	3rd line: crizotinib + trastuzumab → pyrotinib	PR, PLC relieved (symptomatic and radiographic)	10.0	Peritoneum, adrenal gland	Yes	PR	10.0
5	3rd line: crizotinib	PD, PLC deteriorated (symptomatic)	NA	Lung, peritoneum	Yes	PD	0.5
6	2nd line: albumin paclitaxel + capecitabine + apatinib 3rd line: trastuzumab + paclitaxel + oxaliplatin + capecitabine	SD/PR, PLC relieved (radiographic)	5.3/5.0	Lung, brain	No	NA	NA
7	4th line: 5‐FU + oxaliplatin + trastuzumab 5th line: albumin paclitaxel + nivolumab + cisplatin (ip) + endostatin (ip)	PD/PR, PLC relieved (symptomatic and radiographic)	NA/4.3	Lung	No	NA	NA
8	2nd line: albumin paclitaxel + apatinib	SD, PLC stabilized (radiographic)	3.7	Lung	No	NA	NA
9	2nd line: sintilimab + trastuzumab + apatinib	PD, PLC deteriorated (symptomatic and radiographic)	2.0	Lung	No	NA	2.0
10	3rd line: capecitabine + oxaliplatin + irinotecan	NA, PLC relieved (symptomatic)	NP	NA	No	NA	NA
11	1st line: paclitaxel + oxaliplatin + S‐1	PR, PLC relieved (radiographic)	6.7	Lung, LNM	No	NA	NA
12	2nd line: toripalimab + albumin paclitaxel	PD, PLC deteriorated (radiographic)	NA	Adrenal gland	No	NA	1.2
13	5th line: crizotinib	PR, PLC (radiographic)	NA	NA	Yes	NA	NA
14	2nd line: capecitabine + oxaliplatin 3rd line: crizotinib 4th line: volitinib	PD/PR/SD, PLC relieved (radiographic)	NA/3.0/2.0	Lung, primary tumor	Yes	PR	3.0
15	1st line: crizotinib	PR, PLC relieved (radiographic)	14.0	Ovary	Yes	PR	14.0
16	1st line: albumin paclitaxel + S‐1 2nd line: crizotinib	PR/SD, PLC relieved (symptomatic and radiographic)	8.0/3.0	Brain, peritoneum	Yes	SD	3.0
17	2nd line: crizotinib	PR, PLC relieved (radiographic)	6.0	Ovary	Yes	PR	6.0
18	3rd line: crizotinib	PR, PLC relieved (symptomatic, radiographic)	8.0	LNM	Yes	PR	8.0
19	1st line: capecitabine + oxaliplatin 2nd line: albumin paclitaxel	PD/PD, PLC deteriorated (radiographic)	NA	Lung, LNM	No	NA	0.6
20	1st line: albumin paclitaxel + S‐1 + nivolumab	PR, PLC relieved (radiographic)	4.5	Bone	No	NA	NA

Abbreviations: BOR, best of response; ip, intrapleural/intraperitoneal; LNM, lymph node metastasis; NA, not available; NP, not progressed; PD, progressive disease; PFS, progression‐free survival; PLC, pulmonary lymphangitis carcinomatosis; PR, partial response; SD, stable disease; TTP, time to progression.

### 3.5 Analysis of the molecular heterogeneity in MET‐amplified GC

**FIGURE 3:**
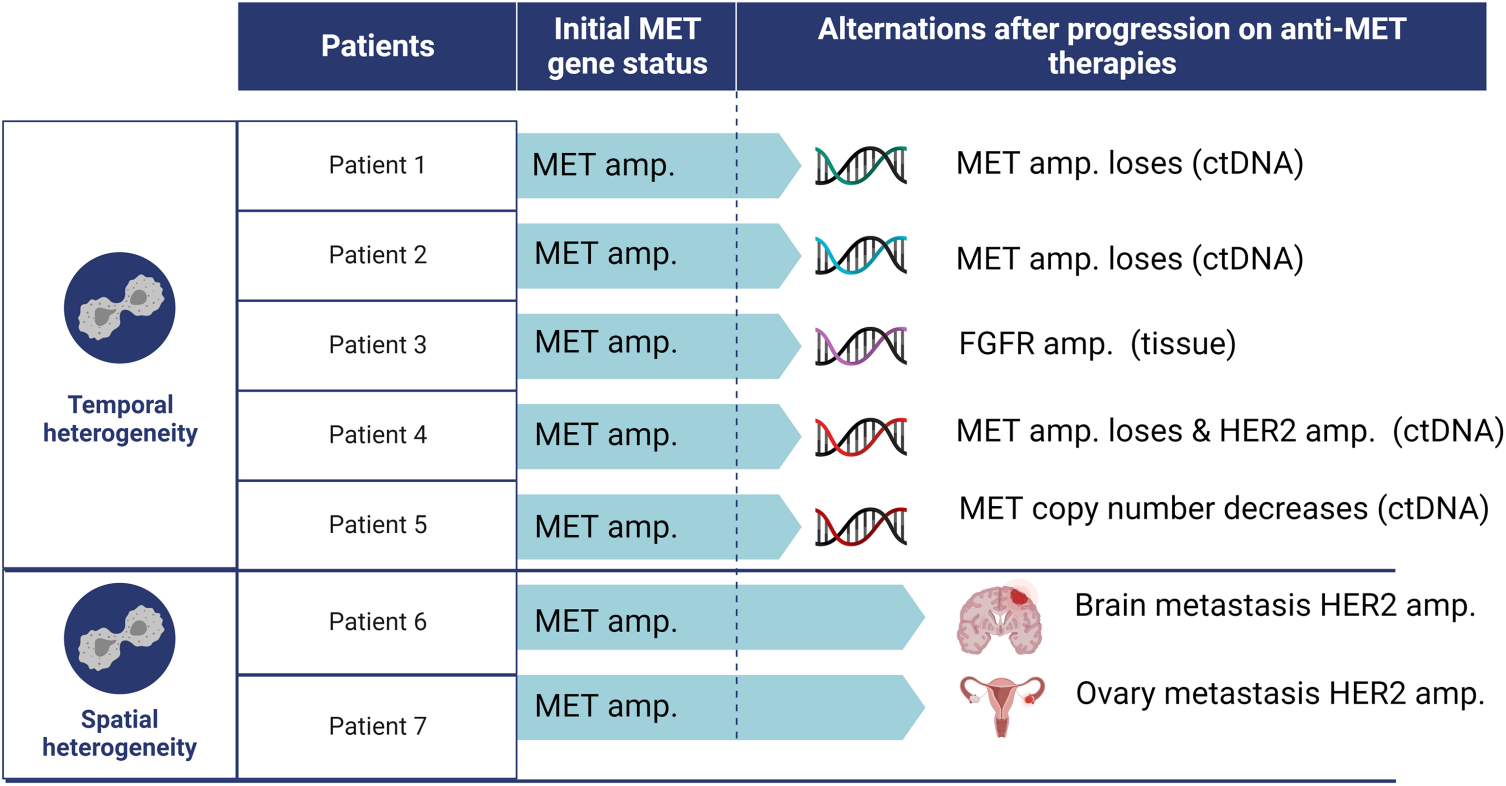
Molecular heterogeneity in MET‐amplified GC.

Temporal and spatial heterogeneity were found in several patients who progressed on anti‐MET therapies (Figure 3). MET copy numbers were sharply reduced in 4 patients. Tumor resampling suggested HER2 and FGFR amplification in two of them, respectively. In addition, one patient with a HER2‐negative primary tumor developed HER2‐positive brain metastasis. Another patient with a HER2‐negative primary tumor developed HER2‐positive ovary metastasis. Both patients received anti‐HER2 therapies and achieved local control of the new‐onset lesions.

## 4 DISCUSSION

MET‐amplified GC is a rare tumor subtype that has shown sensitivity to anti‐MET TKIs in small‐scale studies. However, the reported response rates are diverse. In a phase 2 single‐arm trial, second‐line AMG‐337 (a small‐molecule MET inhibitor) resulted in an overall response rate (ORR) of 18% in patients with pretreated MET‐amplified GC, with a median PFS of 3.4 months.^21^ In the VICTORY umbrella trial, second‐line treatment with savolitinib (a highly selective small‐molecule MET inhibitor) resulted in an ORR of 50% in 20 metastatic MET‐amplified GC patients.^22^ However, in unselected populations, anti‐MET therapies only show suboptimal effects. In molecular unselected GC patients treated with foretinib (an oral, multitarget MET inhibitor), 23% achieved SD with a median duration of 3.2 months.^25^ Among unselected metastatic GC patients treated with tivantinib (a small‐molecule selective MET inhibitor), 36.7% achieved disease control with a median PFS of only 43 days.^26^ These inconsistent results indicate the necessity of precise screening of beneficiaries. It should be emphasized that in real‐world conditions, MET‐amplified GC is typically aggressive or advanced. Patients are commonly assessed with an ECOG PS of 3 or 4 and are generally excluded from trials that recruit participants with fair physical status. Therefore, the authentic efficacy of anti‐MET therapies in GC in the real‐world setting remains unknown.

We report for the first time the clinicopathological and molecular features of MET‐amplified GC. The absence of an internationally recognized set of standards has led to the investigation of various cutoff values to define MET amplification. We took a cutoff of 2.0 (copies in tumor tissue normalized by control) in our study. Our previous research used this cutoff, which mirrored the HER2 amplification cutoff.^11^ In another clinical study that had distinct detection methods and platforms, 6‐copy was used as a cutoff.^23^ To determine the gold standard for MET amplification in GC patients, further studies are definitely required. In our cohort, MET‐amplified GC is highly invasive and aggressive, manifests as multiple organ metastases involving the lung, liver, and ovary, and is often accompanied by serous effusions, which is in line with previous reports.^11^, ^13^, ^27^ Patients with primary MET‐amplified GC had a significantly worse prognosis than those with secondary MET‐amplified GC. This is comprehensible since acquired MET aberrations often occur in the later course of the disease secondary to therapeutic pressure. The biological behavior is similar to that of MET nonamplified tumors before MET amplification develops, but since then, the disease could be aggressive and lethal. In terms of prognostic markers, we found that distant organ metastasis was not associated with prognosis. Our study also suggests that MET amplification could coexist with HER2 positivity but is rarely accompanied by EBV infection or dMMR status. Twenty‐eight patients in our cohort received anti‐MET TKIs, of which 20 (71.4%) achieved disease control. Considering that most patients were heavily pretreated or progressed after multiline therapy, these efficacy data are impressive. In addition, patients who have undergone anti‐MET treatment have superior OS, which also verifies the effectiveness of the treatment. The small sample size might explain the insignificance of the statistical results. Nevertheless, despite the desirable efficacy, the duration of response is often transient, suggesting there is a rapid development of resistance over time in a few cases.

Importantly, in this study, we identified that PLC is a unique manifestation of GC. According to past literature, PLC denotes an end‐stage condition of malignancy characterized by the infiltration of lymphatic vessels by tumor cells. GC with PLC is a rare condition, with only a few cases reported, and the disease characteristics or management have not been extensively investigated.^28^, ^29^ In our cohort, we comprehensively depicted the clinicopathological and molecular patterns of GC with PLC. Compared to GC without PLC, GC with PLC has earlier ages at onset, higher tumor burden, more tumor spreading or dissemination, and worse ECOG PS. Patients are inclined to develop compression symptoms due to pleural or peritoneal effusions. No molecular marker (including TMB) alone was found to be significantly associated with the prognosis of patients with PLC, which warrants future investigations in a large‐scale cohort. It is surprising that patients with PLC did not have a worse prognosis. The possible explanations are as follows: (1) PLC was a predictor of poor prognosis in the univariate analysis, but not in the multivariate analysis, indicating a potential indirect association between PLC and the prognosis of patients with MET‐amplified GC; (2) In our study, we performed Cox regression analysis on 58 patients with MET‐amplified GC who themselves had a poor prognosis, as opposed to unselected patients. It is assumed that MET amplification, not PLC, is the determinant of a worse prognosis.

**FIGURE 4:**
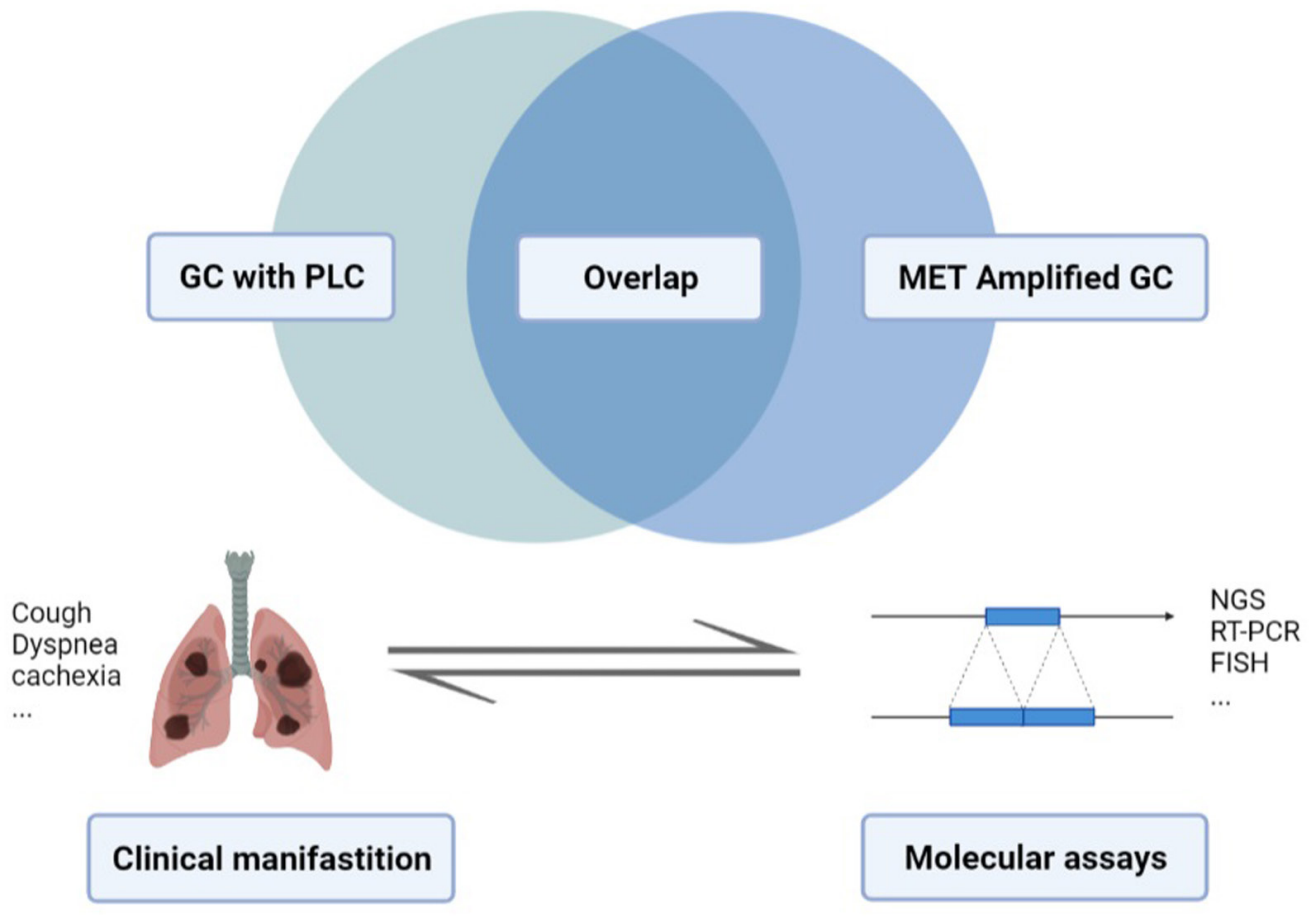
Association between PLC and MET gene amplification.

It is noteworthy that GC with PLC accounts for a considerable proportion of MET‐amplified GC. In our cohort, 16/58 (27.6%) patients developed PLC at any point during the disease course. Among 17 patients with GC and PLC who had assessable MET status, 13 (76.5%) were confirmed to be MET‐amplified, indicating that PLC is enriched in MET‐amplified tumors. It is reasonable to infer that aberrant MET signaling potentially gives rise to PLC, which confers profound clinical implications. In this regard, a patient with PLC should be suspected of having a MET‐amplified tumor and be referred for genetic testing. Conversely, clinicians should be alert to PLC during the subsequent course of MET‐amplified GC for earlier interventions (Figure 4). Given the rarity of MET amplification and the economic cost of molecular testing, it is key to choose an appropriate screening time. According to published reports, acquired MET amplification confers anti‐HER2 resistance in patients with EGFR‐ or HER2‐amplified GC.^30^, ^31^ MET overactivation also mediates resistance to anti‐FGFR therapies.^32^ Our present study suggests that patients who are resistant to multiple treatments or with extensive organ metastasis (especially PLC) are populations suitable for targeted screening.

In our study, we also revealed that systemic therapies often lead to focal control of lung lesions. For patients with MET‐amplified GC, anti‐MET TKIs are remarkably efficient, indicated by target lesion shrinkage and/or rapid symptom relief. A vast number of patients with PLC do not have antitumour opportunities due to a poor physical state (e.g., requiring respiratory support). Nevertheless, once PLC is controlled, patients might regain the opportunity for systemic therapies to diminish tumor burden, therefore improving long‐term survival. In that case, timely molecular diagnosis is crucial. Considering the poor physical condition of patients with PLC, reexamination of tumor tissue is impractical. Liquid biopsy (circulating tumor DNA or pleural/peritoneal fluid DNA sequencing) might be an ideal alternative due to its low invasiveness.

We also shed light on the heterogeneity of MET status in GC. MET aberrations could coexist with other driver molecular changes simultaneously, such as FGFR or HER2 amplification. Previous literature has demonstrated inconsistencies in molecular characteristics between primary and metastatic lesions.^33^, ^34^ Targeted therapies could impose selection pressure on evolving tumors, resulting in disease progression via the outgrowth of MET‐nonaddicted constituents. Thus, molecular heterogeneity might account for treatment resistance. To counteract this, circulating tumor DNA sequencing could be used to longitudinally monitor molecular alterations. In our cohort, MET copy numbers were reduced in 5 crizotinib‐resistant patients, and a novel RTK target was detected in one patient, which suggests the necessity of tumor rebiopsy to assist in clinical decision making.

Our study has several limitations. First, the small‐scale and retrospective nature of this study could be an inherent defect. We acknowledge that it is difficult to interpret the survival data due to the limited number of enrolled patients and the variations in treatments. Further validation is warranted to consolidate the relationship between PLC and MET amplification, although the overall rarity and aggressive nature of GC with PLC make prospective studies challenging. Second, each detection method of MET amplification has its flaws. NGS is less sensitive than FISH, which might affect the detection rate of MET amplification. Third, in our cohort, PLC was diagnosed via CT and clinical manifestations, which might lead to underdiagnosis of very mild cases (subclinical or radiographically invisible cases).

## 5 CONCLUSIONS

Our study indicates that MET amplification could be enriched in gastric cancer accompanied by pulmonary lymphangitis carcinomatosis, and the latter shows a high response rate to anti‐MET target therapies.

## AUTHOR CONTRIBUTIONS

**Zhening Zhang:** Conceptualization (equal); formal analysis (equal); investigation (equal); writing – original draft (equal). **Yiyi Yu:** Conceptualization (equal); data curation (equal). **Tong Xie:** Resources (equal). **Changsong Qi:** Resources (equal). **Xiaotian Zhang:** Resources (equal). **Lin Shen:** Funding acquisition (equal); writing – review and editing (equal). **Zhi Peng:** Conceptualization (equal); funding acquisition (equal); writing – review and editing (equal).

## FUNDING INFORMATION

This work was supported by National Natural Science Foundation of China (81602057); Beijing Natural Science Foundation (Z210015); Clinical Medicine Plus X‐Young Scholars Project of Peking University (PKU2019LCXQ020, PKU2018LCXQ018) and the Fundamental Research Funds for the Central Universities.

## CONFLICT OF INTEREST STATEMENT

The authors declare that they have no competing interests.

## ETHICS STATEMENT

The present study was approved by the ethics committee of Peking University Cancer Hospital (Approval number 2022KT53) and Fudan University Zhongshan Hospital (Approval number 2020253R2), and conducted in accordance with the Declaration of Helsinki. Written informed consent for the participation of this retrospective study was exempted by the ethics committee of Peking University Cancer Hospital and Fudan University Zhongshan Hospital.

## Supporting information


Figure S1.

Figure S2.

Figure S3.

Figure S4.

Figure S5.

Table S1.

## Data Availability

The original data in the current study are available from the corresponding author on reasonable request.
