# Supplementary figures and images for: Pulmonary lymphangitis carcinomatosis: A peculiar presentation clustering in MET‐amplified gastric cancer
Source: Cancer Med. 2023 Sep 29;12(19):19583–94. doi: 10.1002/cam4.6575 (PMC10587944; doi:10.1002/cam4.6575)

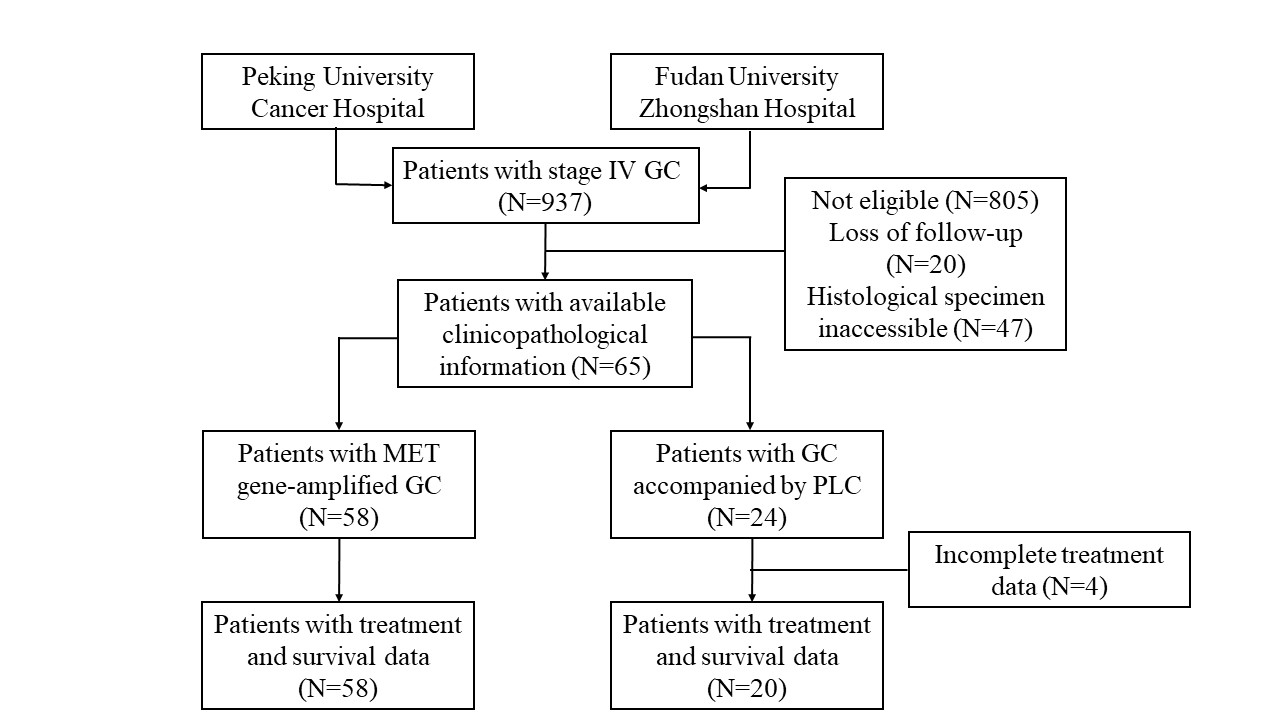

Supplement: Supplementary file 1 — Figure S1. [file CAM4-12-19583-s004.jpg]

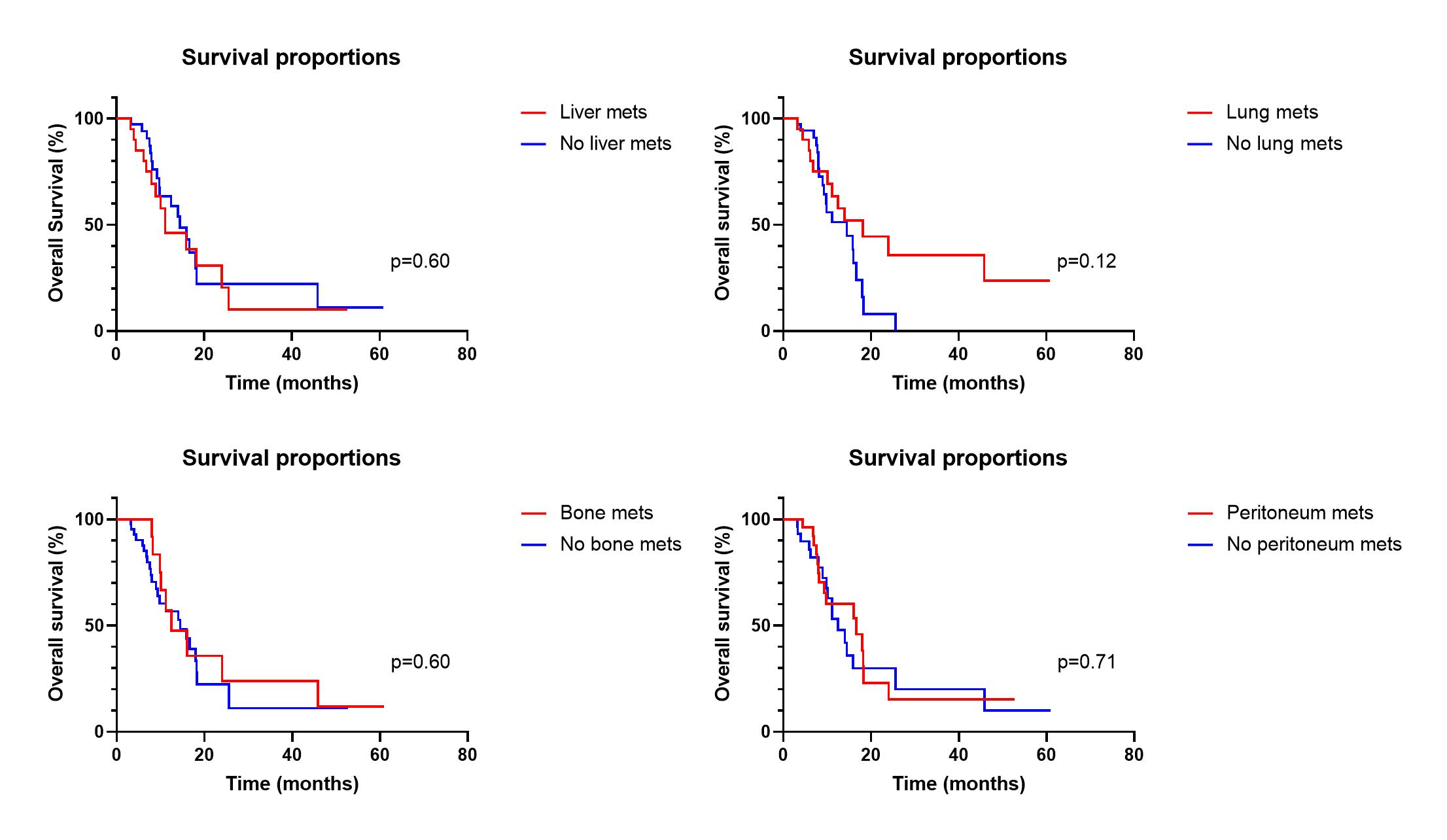

Supplement: Supplementary file 2 — Figure S2. [file CAM4-12-19583-s006.jpg]

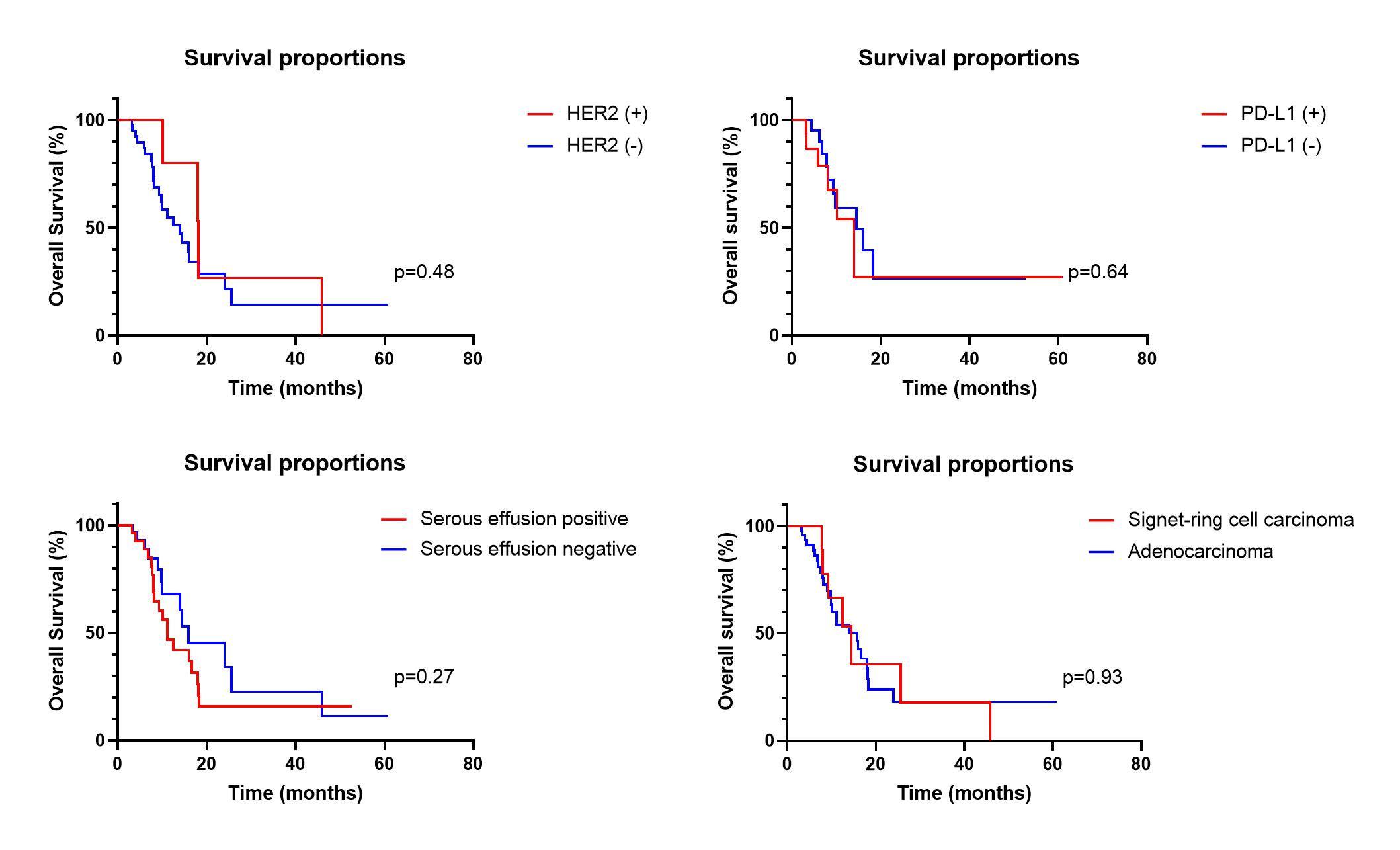

Supplement: Supplementary file 3 — Figure S3. [file CAM4-12-19583-s005.jpg]

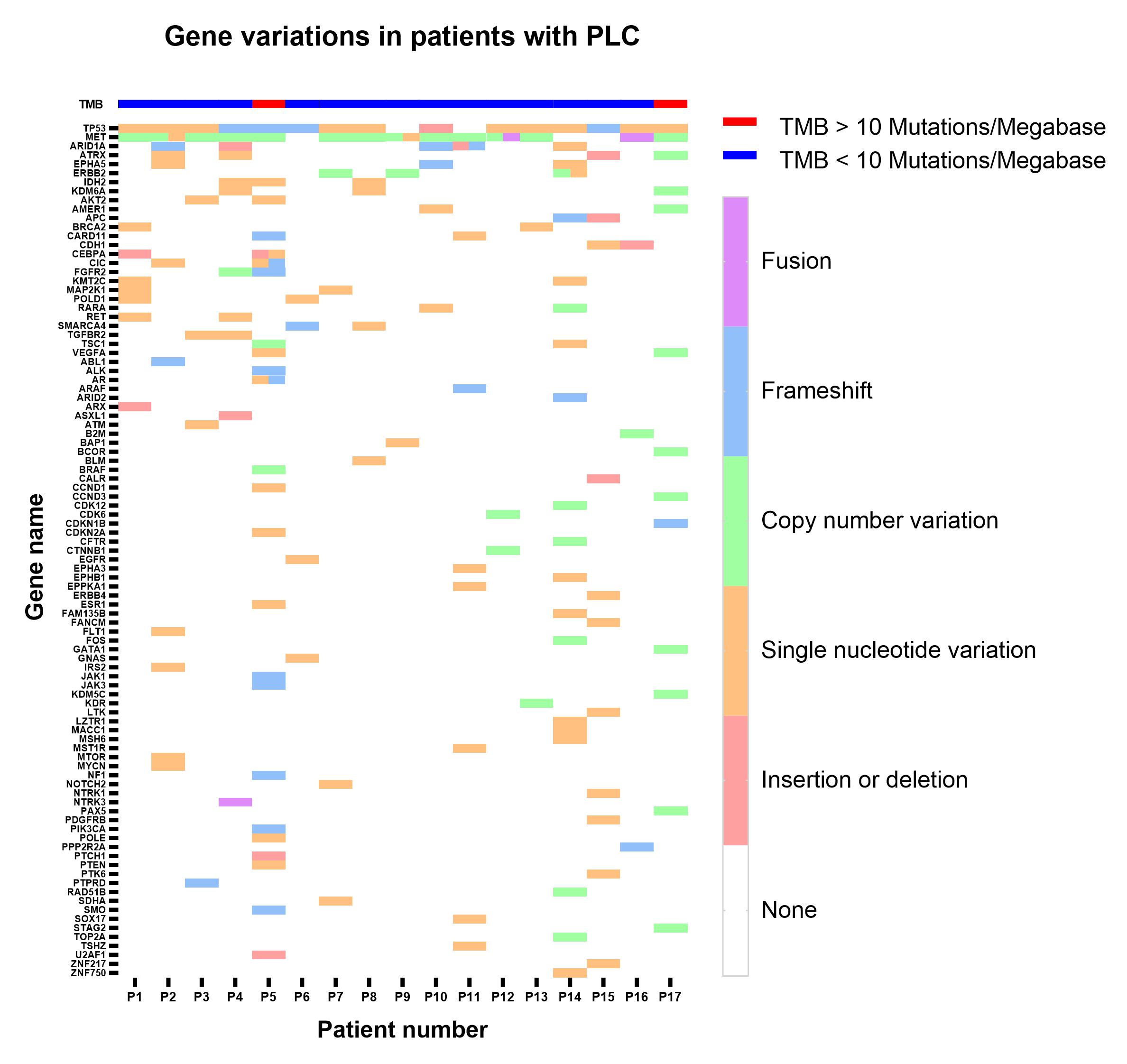

Supplement: Supplementary file 4 — Figure S4. [file CAM4-12-19583-s003.jpg]

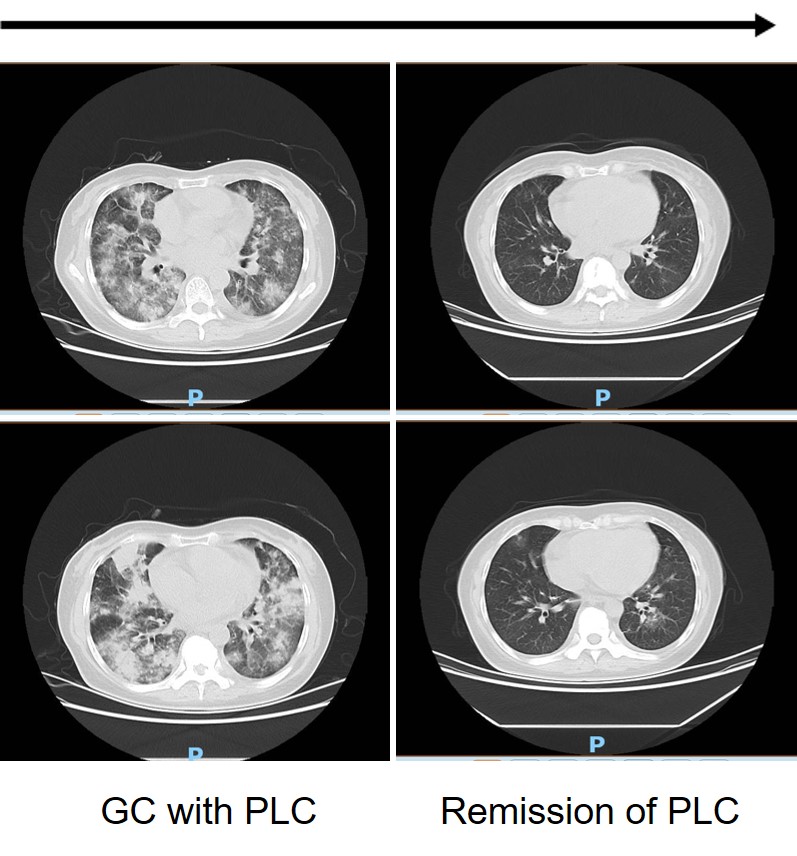

Supplement: Supplementary file 5 — Figure S5. [file CAM4-12-19583-s002.jpg]
