# Supplementary material for: Pulmonary lymphangitis carcinomatosis: A peculiar presentation clustering in MET‐amplified gastric cancer
Source: Cancer Med. 2023 Sep 29;12(19):19583–94. doi: 10.1002/cam4.6575 (PMC10587944; doi:10.1002/cam4.6575)
Supplement: Supplementary file 6 — Table S1. [file CAM4-12-19583-s001.docx]

**Table S1. Clinical and molecular characteristics of patients with MET-amplified GCs according to PLC**

|  | Total cases  (n=58) | MET-amplified GC with PLC  (n=13) | MET-amplified GC without PLC  (n=45) | P value |
| --- | --- | --- | --- | --- |
| Variables |  |  |  |  |
| Age (year) | 53 | 44 | 56 | 0.002 |
| Sex |  |  |  | 0.010 |
| Male | 37 | 4 | 33 |  |
| Female | 21 | 9 | 12 |  |
| ECOG PS |  |  |  | 0.016 |
| 0-1 | 44 | 7 | 37 |  |
| ≥2 | 14 | 6 | 8 |  |
| Primary tumor site |  |  |  | 0.318 |
| Cardia | 12 | 1 | 11 |  |
| Body | 26 | 7 | 19 |  |
| Pylorus | 20 | 5 | 15 |  |
| Histological type |  |  |  | 0.439 |
| Adenocarcinoma | 48 | 10 | 38 |  |
| Signet-ring cell carcinoma | 10 | 3 | 7 |  |
| Lauren classification |  |  |  | 0.171 |
| Intestinal | 14 | 2 | 12 |  |
| Mixed/Diffuse | 44 | 11 | 33 |  |
| Differentiation |  |  |  | 0.084 |
| Poorly differentiated | 44 | 12 | 32 |  |
| Moderate/Well-differentiated | 14 | 1 | 13 |  |
| Serous effusion |  |  |  | 0.026 |
| No effusion | 30 | 5 | 25 |  |
| Only hydrothorax | 19 | 4 | 15 |  |
| Only hydroperitoneum | 5 | 2 | 3 |  |
| Hydrothorax and hydroperitoneum | 4 | 2 | 2 |  |
| Liver metastasis |  |  |  | 1.000 |
| Yes | 21 | 5 | 16 |  |
| No | 37 | 8 | 29 |  |
| Bone metastasis |  |  |  | 0.091 |
| Yes | 15 | 5 | 10 |  |
| No | 43 | 8 | 35 |  |
| Lung metastasis |  |  |  | 0.000 |
| Yes | 21 | 11 | 10 |  |
| No | 37 | 2 | 35 |  |
| HER2 status |  |  |  | 0.343 |
| Positive | 7 | 1 | 6 |  |
| Negative | 51 | 12 | 39 |  |
| MMR status |  |  |  | NA |
| pMMR | 58 | 13 | 45 |  |
| dMMR | 0 | 0 | 0 |  |
